# Functional complexity of the *Leishmania* granuloma and the potential of *in silico* modeling

**DOI:** 10.3389/fimmu.2013.00035

**Published:** 2013-02-15

**Authors:** John W. J. Moore, Daniel Moyo, Lynette Beattie, Paul S. Andrews, Jon Timmis, Paul M. Kaye

**Affiliations:** ^1^Centre for Immunology and Infection, Hull York Medical School and Department of Biology, University of YorkYork, UK; ^2^Department of Computer Science, University of YorkYork, UK; ^3^Department of Electronics, University of YorkYork, UK

**Keywords:** granuloma, leishmaniasis, visceral, inflammation, *in silico* modeling, imaging

## Abstract

In human and canine visceral leishmaniasis and in various experimental models of this disease, host resistance is strongly linked to efficient granuloma development. However, it is unknown exactly how the granuloma microenvironment executes an effective antileishmanial response. Recent studies, including using advanced imaging techniques, have improved our understanding of granuloma biology at the cellular level, highlighting heterogeneity in granuloma development and function, and hinting at complex cellular, temporal, and spatial dynamics. In this mini-review, we discuss the factors involved in the formation and function of *Leishmania donovani*-induced hepatic granulomas, as well as their importance in protecting against inflammation-associated tissue damage and the generation of immunity to rechallenge. Finally, we discuss the role that computational, agent-based models may play in answering outstanding questions within the field.

## INTRODUCTION

Visceral leishmaniasis (VL), a parasitic disease impacting on health and economy in developing countries, is caused by *Leishmania donovani* and *L. infantum*. These parasites establish long-term infection within multiple organs including the spleen, liver, and bone marrow. In humans and dogs, VL is invariably fatal if untreated, but subclinical infections are common and are associated with granuloma formation (Pearson and Sousa, 1996; Sanchez et al., 2004). In experimental VL (EVL) in mice, granuloma formation is associated with self-limiting hepatic infection, whereas granulomas fail to form in spleen, where parasites persist (Murray, 2001). Together, these observations suggest a causal association between granuloma formation and host resistance to visceralizing species of *Leishmania*.

**FIGURE 1:**
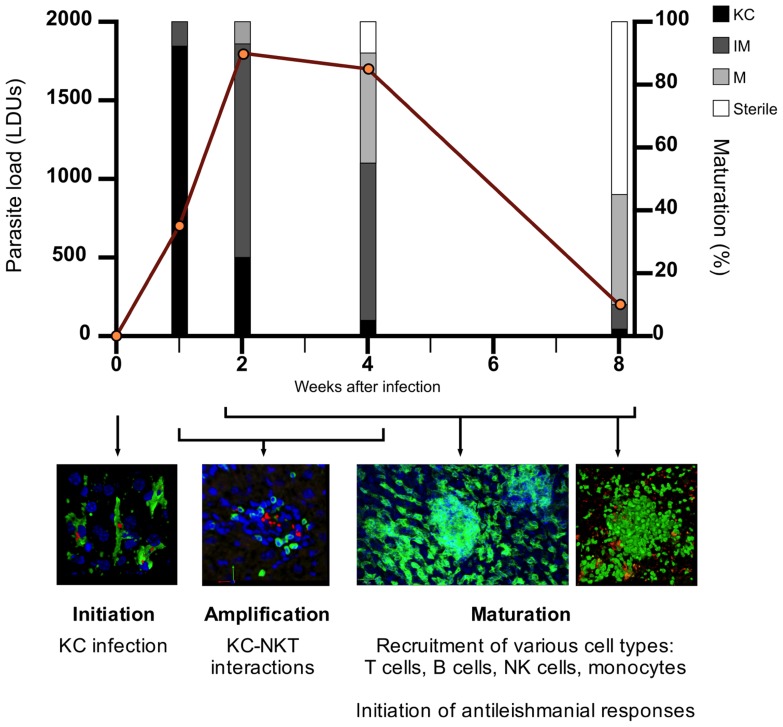
**The process of granuloma maturation during *L. donovani* infection**. The infection of resident liver macrophages (Kupffer cells; KCs) initiates the process of granulomatous inflammation (*initiation*). Hepatic NKTs migrate toward infected KCs and their interaction triggers the recruitment of mononuclear cells to the liver (*amplification*). Various cell types, predominantly T cells, are recruited to infected KC foci within the liver, with granulomas developing in size and cellularity in an asynchronous manner over the first 4 weeks post-infection (p.i.) (*maturation*). During the *maturation* stage, the inflammatory response peaks around 4 weeks p.i. where the antileishmanial response becomes sufficient to begin clearing the parasite burden. As parasites are cleared from individual infected KC foci, cells begin to move out of the granuloma, returning the liver to its original pre-infection state. Hepatic granulomatous inflammation resolves by 8 weeks p.i. with the majority of parasites cleared. Data on parasite load and granuloma maturation redrawn from Murray (2001). KC, Kupffer cell; IM, immature granuloma; M, mature granuloma; sterile, sterile granuloma.

Granulomas progress through distinct stages of “maturation,” as described in **Figure 1**. Fundamental insights into the role of different immune cells, effector and regulatory cytokines, and other mediators have been made through the use of gene-targeted mice (reviewed in Murray, 2001; Kaye et al., 2004; Stanley and Engwerda, 2007). However, the approach of using knockout (KO) mice or blocking/depleting antibodies is limited when asking questions about immune regulation within these discrete inflammatory foci. In this mini-review, we will attempt to link recent studies involving direct visualization of hepatic granulomas with previous findings, discuss how the granuloma may function at a cellular and spatiotemporal level and highlight important unanswered questions. We also discuss the potential of *in silico* modeling to aid our understanding of these fascinating structures.

## THE DYNAMIC MICROENVIRONMENT OF THE GRANULOMA

### KC–NKT CELL INTERACTIONS AND T CELL RECRUITMENT

In EVL, Kupffer cells (KCs) are at the heart of the hepatic granuloma, often fusing with each other as a result of migration from neighboring sinusoids (Murray et al., 1987; Beattie et al., 2010a). The signals that trigger KC migration remain to be identified. The KC-rich core acts as a platform for the recruitment of other cells, notably T cells and monocytes (Beattie et al., 2010a). The exact mechanisms resulting in cell recruitment are unknown, although blood flow, adhesion molecules (Murray, 2000; Engwerda et al., 2004), chemokines (Sato et al., 1999), and cytokines such as interleukin (IL)-1 (Curry and Kaye, 1992) have all been implicated.

Invariant natural killer T cells (iNKTs) play a significant amplifying role. iNKTs-deficient mice have impaired granuloma maturation, lower inflammatory cytokine expression, and reduced expression of CCL2, CXCL5, and CXCL2 (Robert-Gangneux et al., 2012). iNKTs interact with KCs via a signal regulatory protein alpha-CD47 dependent amplification loop to regulate the production of the T cell-chemoattractant CXCL10 (Svensson et al., 2005; Beattie et al., 2010b), shown to be host protective in EVL (Gupta et al., 2009). KC–NKT interactions also feature in other infection models (Lee et al., 2010), but the consequences of NKT cell activation may not always be favorable. For example, treatment with α-galactosylceramide, an activator of iNKTs, decreased rather than enhanced resistance (Stanley et al., 2008). It remains to be directly shown whether iNKTs are retained within granulomas but cxcr6^gfp/^^+^ mice (Geissmann et al., 2005) could be used to address this question.

### T CELLS: STRENGTH IN NUMBERS?

T cells are the predominant cell type present within the *L. donovani*-induced granuloma and granuloma maturation (**Figure 1**). Both CD4^+^ and CD8^+^ T cells are indispensable in the effective formation and function of granulomas and for parasite clearance (Stern et al., 1988). 4D intravital imaging studies in EVL and in mice with bacille Calmette–Guérin (BCG) infection indicate that T cells move relatively freely within granulomas, with no requirement for antigen-specificity for granuloma entry (Beattie et al., 2010a; Egen et al., 2011). Antigen presentation within granulomas appears limited in both these models. Of note, during *L. major* infection the effector function of a few antigen-specific CD4^+^ T cells is enhanced by bystander activation (Muller et al., 2012). We have found that ~70% of CD4^+^ T cells within infected livers display an activated phenotype (CD44^hi^), with ~30–40% of CD4^+^ T cells having the capacity to produce interferon-gamma (IFN-γ; manuscript submitted). These data are consistent with a model whereby local bystander activation operates within (and possibly even between) granulomas to enhance effector function. Future studies should aim to test such a model and determine whether non-specific T cell recruitment is beneficial for the outcome of *Leishmania* infection. Furthermore, the extent to which T cell functional differentiation occurs within the granuloma environment remains open.

### THE ROLE OF GRANULOMA-ASSOCIATED MONOCYTES

Monocytes are present in *L. donovani*-induced granulomas but do not appear to be infected. In contrast, both infected and uninfected monocytes are recruited to BCG-induced hepatic granulomas (Egen et al., 2008). In a zebrafish embryo model, monocytes were recruited to *Mycobacterium avium*-induced granulomas throughout infection, and were shown to be capable of becoming infected, migrating away from the granuloma, and establishing infection in distant sites (Davis and Ramakrishnan, 2009). It is important to note that zebrafish embryos lack T cells (Willett et al., 1999; Trede et al., 2001) but, nonetheless, this suggests an interesting link between granuloma formation and disease dissemination. To date, we have no evidence that monocytes behave in this way during EVL. The blocking of monocyte recruitment to *L. donovani*-induced granulomas using anti-type 3 complement receptor (CR3) antibody resulted in impairment of early antileishmanial resistance followed by delayed granuloma maturation (Cervia et al., 1993). This indicates the importance of monocytes in effective granuloma formation but we do not fully understand how the monocytes carry out this function. Similarly, it is unknown if a proportion of macrophages in the granuloma core represents differentiated migratory monocytes. Inflammatory monocytes are capable of migrating from the blood and promoting inflammation (Shi and Pamer, 2011). Thus, it would be informative to determine the phenotype of granuloma monocytes to best understand their ability to propagate the liver inflammatory response.

CD11c^+^ dendritic cells (DCs) are also found within *L. donovani*-induced granulomas although they were shown not to be the targets of effector CD8^+^ T cells (Beattie et al., 2010a). In addition, during BCG infection, CD11c^+^ cells in chronic granulomas displayed decreased expression of major histocompatibility complex (MHC) class II and co-stimulatory molecules whereas CD11c^+^ cells within acute granulomas could support the reactivation of recruited antigen-specific CD4^+^ T cells and could induce IFN-γ responses from naïve T cells (Schreiber et al., 2010). The role of DCs in antigen presentation to CD4^+^ T cells in granulomas during EVL remains to be addressed.

### BALANCE BETWEEN INFLAMMATION AND TISSUE DAMAGE

We have shown B cells to be recruited to granulomas throughout hepatic infection, and although antigen-specificity did not affect their recruitment, cognate B–T interactions could take place (Moore et al., 2012). We found no direct evidence of regulatory B cells, though these cells have been described by others in EVL (Deak et al., 2010), but nevertheless B cell-deficient mice have accelerated granuloma formation (with neutrophil infiltration) and enhanced parasite clearance during EVL (Smelt et al., 2000). B cells, therefore, play a role in preventing liver pathology, via the control of neutrophil infiltration, highlighting a divorce between the control of parasite burden and the induction of tissue pathology.

Liver pathology is also observed in the case of tumor necrosis factor (TNF) deficiency where high liver parasite burdens do not result in liver pathology until the severely delayed and exaggerated inflammatory response begins, resulting in hepatic necrosis (Murray et al., 2000). However, in contrast to other experimental models of hepatic inflammation and infection, such as schistosomiasis, EVL in wild type (WT) mice does not appear to induce overt liver pathology or fibrosis. The T helper type 1 (Th1)-dominated inflammatory response observed during EVL is not normally associated with fibrosis-initiating mechanisms. However, there is evidence suggesting hepatic fibrosis can occur in EVL, which is particularly relevant to human VL where more extensive fibrosis has been described (el Hag et al., 1994; Duarte et al., 2009). Hepatic fibrosis occurs in a range of chronic inflammatory conditions, and is typically triggered by the activation of hepatic stellate cells (HSCs) and the transition of stellate cells into myofibroblasts, resulting in deposition of collagen and other extracellular matrix proteins (Gressner and Bachem, 1995). Alternatively activated macrophages (AAM) are also implicated in the regulation of fibrosis (Wynn and Barron, 2010), and AAM-associated cytokines are implicated in the hepatic response during EVL. IL-13-deficiency affects granuloma maturation, but reports differ in the extent to which this affects parasite clearance (Murray et al., 2006; McFarlane et al., 2011). IL-4 KO and IL-4R KO mice also displayed delayed granuloma maturation, suggesting a role for IL-4 in the hepatic inflammatory response to *L. donovani* infection (Stager et al., 2003). Of interest, in mice co-infected with *L. donovani* and *S. mansoni*, hepatic granulomas fail to form around *L. donovani*-infected KCs in the vicinity of egg granulomas where AAMs are abundant, whereas *L. donovani*-infected KCs in the parenchyma do serve as a focus for granuloma maturation (Hassan et al., 2006). Further studies should aim to elucidate the function of HSCs and AAMs within the liver during EVL, and may help to create mouse models that are more representative of human disease.

The occurrence of overt pathology in various mouse KO models of EVL suggests that processes are present in WT mice to protect against inflammation-induced hepatic tissue damage. In addition to a regulatory role for B cells in EVL, natural killer (NK) cells have been shown to be present within *L. donovani*-induced granulomas and displayed immunoregulatory properties (Maroof et al., 2008). However, this study did not address whether the loss of NK cells led to enhanced liver pathology.

Whether regulatory T cells (Tregs) are recruited to *L. donovani*-induced granulomas and/or protect against pathology is unknown and is an important question to address. We have shown ~2–3% of CD4^+^ T cells within infected livers co-express IFN-γ and IL-10, indicative of a regulatory phenotype (manuscript submitted). Cytotoxic T lymphocyte-associated antigen 4 (CTLA-4) is expressed by Tregs and is important for executing their immunosuppressive function (Read et al., 2000; Takahashi et al., 2000). We have shown that using anti-CTLA-4 blocking antibody during EVL enhanced granuloma maturation and parasite killing (Zubairi et al., 2004), one explanation for this result being that Tregs are operating to limit granulomatous inflammation. Whether this limitation protects against excessive inflammation and the development of tissue pathology is an interesting question to address. Granuloma-derived transforming growth factor-β (TGF-β) also inhibited IFN-γ production by CD4^+^ T cells during EVL (Wilson et al., 1998), providing further evidence that regulatory mechanisms are operating with *Leishmania*-induced granulomas. Studies in schistosomiasis-associated granulomas demonstrated a role for regulatory CTLA-4^+^ CD25^-^ CD4^+^ T cells in protection against host mediated pathology, with blocking of these Tregs resulting in significant weight loss but enhanced recruitment of effector T cells to hepatic granulomas (Walsh et al., 2007). Other studies have demonstrated the presence of Tregs within pulmonary granulomas, with Tregs being found in both *Mycobacterium tuberculosis* (*Mtb*) infection (Scott-Browne et al., 2007) and the autoimmune disorder sarcoidosis (Taflin et al., 2009). Understanding the balance between immunoregulation and inflammation-induced tissue damage in greater detail will prove crucial in optimizing future therapeutic approaches designed at enhancing the inflammatory response to infection.

Regulatory cells within *L. donovani*-induced granulomas may also function to control the resolution stage of hepatic granuloma formation. As describe in **Figure 1**, granuloma maturation peaks around 4 weeks post-infection (p.i.) followed by parasite clearance and the resolution of granulomas, enabling the liver architecture to return to its pre-infection state. As the hepatic granuloma response continues to increase in the first 4 weeks of infection, it would be reasonable to posit that there must be a mechanism(s) in place to act as “a brake” on the immune response and begin the resolution stage of EVL. Whether the various cell types mentioned in the previous section play a role in this is an interesting topic that has not been previously addressed.

### IMMUNITY

A final theme that has received little attention is whether or not granulomas are essential for the promotion and/or maintenance of memory required for immunity to reinfection. Secondary protection relied on CD8^+^ T cells and resulted in accelerated granuloma development at infected foci (Murray et al., 1992). Interestingly, treatment with cyclosporin A during reinfection resulted in an absence of granuloma formation but did not affect protection (Murray et al., 1992). These data provides compelling evidence that granuloma formation may be dispensable in providing protection against *L. donovani* infection. However, to date, there has been no study of rechallenge that did not result in granuloma formation during the primary challenge. Thus, it is still unknown whether granuloma formation plays a role in generating memory responses and this question will be critical in addressing whether hepatic granulomas are indeed indispensable to immunity during EVL.

## MODELING GRANULOMAS

### THE POTENTIAL OF *IN SILICO* MODELING

Understanding the complex inner workings of the granuloma microenvironment is challenging. *Ex vivo* studies only provide experimental observations at individual snapshots in time and without blood perfusion, vital nutrients are lost and the liver sinusoidal structure and its contents become disrupted, leading to a rapid impairment of *ex vivo* liver function. Although manipulation of intact mice may alter granuloma form and function, such interventions also target events outside the granuloma microenvironment. *In silico* models can avoid these caveats and provide novel insight into systems through predictive modeling, providing time-course data and allowing an iterative process of hypothesis testing and experimental validation. This cycle of experimentation and validation can prove useful for ascertaining effects of experimental interventions.

Computational modeling of the dynamics of granuloma formation during *Mtb* infection (Segovia-Juarez et al., 2004; Gammack et al., 2005; Fallahi-Sichani et al., 2011) has helped elucidate the role of CD8^+^ T cell effector function (Sud et al., 2006), DC trafficking and antigen presentation (Marino et al., 2004) and of TNF in granuloma maintenance (Ray et al., 2009; Fallahi-Sichani et al., 2010; Marino et al., 2010). These studies highlight the need to move from a more traditional reductionist study of biological systems, toward creating an “integrative picture of a system” using multi-scale modeling to capture system behaviors across both biological- and temporal-scales (Kirschner et al., 2007). Multi-scale modeling often integrates various modeling approaches, such as agent-based modeling (ABM) and equation-based modeling. In this review, we focus only on ABM.

### AGENT-BASED MODELING OF GRANULOMAS IN THE LEISHMANIASES

Modeling granuloma formation in the leishmaniases is in its infancy, and our efforts have focused on ABM as a suitable technique for investigating granuloma formation, structure, and function. ABMs can be used to extend equation-based modeling approaches to account for spatiotemporal system dynamics (Germain et al., 2011). However ABMs can also be created and used as stand-alone simulation software. ABMs are typically built from behaviors and data derived from *in vivo* experimentation to explore hypothesized behaviors of cellular populations and cellular interactions.

**FIGURE 2:**
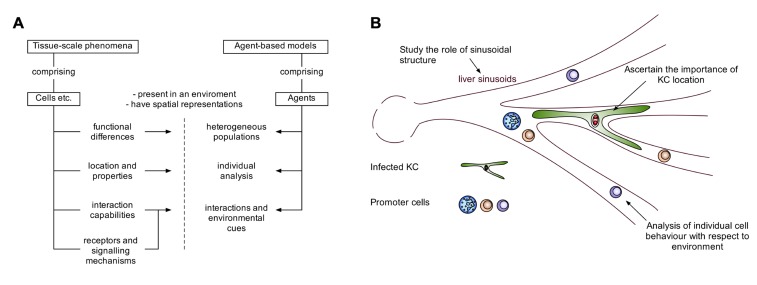
**The potential of agent-based modeling for modeling tissue-scale phenomena**. ABMs allow the acquisition of time-course data with a custom degree of granularity. **(A)** A schematic demonstrating how features of entities such as cells in tissue-scale phenomena, can be captured by features of agent-based models. **(B)** Illustrating the advantages of agent-based modeling in the context of hepatic granuloma formation.

Several studies have made use of ABM to study various forms of inflammation (Li et al., 2008; Dong et al., 2010; Brown et al., 2011), and a recent review has highlighted the benefits and challenges of the approach for inflammatory contexts (An and Christley, 2012). Indeed, ABMs have various advantages for studying inflammation and tissue-scale phenomena (**Figure 2A**). The study of granuloma formation is invariably linked to both the structure of the granuloma, and the spatial environment in which the granuloma is present. ABM allows for spatial representation of agents (e.g., cells, molecules), in addition to functional abilities/states for each agent (e.g., rate of cytokine production, receptor expression), providing the potential to compare biophysical and functional mechanisms. These models can represent agents individually with their own state providing heterogeneity within a population of agents, allowing for the study of small differences in individual agent behavior that manifest at the population level. Individual agent representation also provides the ability to interrogate and track agents. For example, this facilitates the study of cell recruitment into a granuloma, with respect to the surrounding environment, and the analysis of various interactions and environmental cues received by those cells, resulting in a powerful predictive tool (**Figure 2B**). The choice of ABM approaches should always match the purpose for the model. For example, modeling large populations of homogenous cells or molecules to study population level properties may lend itself more, in terms of efficiency, to mathematical approaches, although frameworks to efficiently model large homogenous populations with ABMs do exist (Holcombe et al., 2006).

Our initial *in silico* studies focused on developing tools to understand the well-documented heterogeneity of the granulomatous response that leads to fully formed mature granulomas residing side by side with infected KCs showing minimal inflammatory cell recruitment (**Figure 1**). This ABM provided a means for evaluating whether granuloma heterogeneity might reflect competition for NKT cells or KC diversity (Flugge et al., 2009), but was limited from a computational and biological perspective. A more complex stochastic Petri-net model investigated novel therapeutic interventions for host protection during *L. donovani* infection (manuscript submitted). This model suggests individual granulomas have a heterogeneous capacity for parasite clearance, and predicts that KC autocrine IL-10 is a key regulator of intra-granuloma effector mechanisms. Recently, we have developed a tissue-scale ABM to investigate the dynamics of NKT cell recruitment and KC stimulation during early infection, providing insight into interventions for the promotion of an early granulomatous response. Our model uses artificial sinusoidal environments, generated using published imaging and statistical analysis data (Hoehme et al., 2010) and demonstrates the importance of liver architecture in granuloma positioning.

## CONCLUSION

*Leishmania donovani*-induced granulomas represent an intriguing example of innate and adaptive immunity combining in a unique microenvironment to eradicate an intracellular pathogen. Each granuloma represents a highly organized structure and studies discussed in this review have shown that several different cell types and factors function within this complex structure to deliver effective parasite clearance, without causing excessive tissue damage. Interestingly, it is still unknown if granuloma formation is indispensable for immune protection but granulomatous inflammation likely provides the most efficient mechanism of delivering key antileishmanial processes in a focused manner. The advancement of imaging techniques has allowed us to study granulomas in greater detail, highlighting the dynamic and complex nature of these microenvironments, but as a result have raised important questions. The ability of ABMs to create an integrated system, incorporating the multiple factors involved in granuloma formation, offers a powerful tool to help address these questions, enabling the testing of hypotheses and discovery of potential interventions. Future studies should aim to use a combination of experimentation and modeling to unearth the complexities of the *Leishmania* granuloma and discover how exactly this response delivers its effective antileishmanial function.

## Conflict of Interest Statement

The authors declare that the research was conducted in the absence of any commercial or financial relationships that could be construed as a potential conflict of interest.
